# Qualitative Research—What You Need to Know (For Now)

**DOI:** 10.1111/aas.70157

**Published:** 2025-11-24

**Authors:** Natalie Buu, Thomas Engelhardt

**Affiliations:** ^1^ Department of Anaesthesia Montreal Children's Hospital, McGill University Health Centre Montréal Quebec Canada; ^2^ Institute of Health Sciences Education McGill University Montréal Quebec Canada

There is a new kid on the block: qualitative research. Actually, it is not very new but to clinicians dealing with numbers, equations, cause and effect, associations and ever more elaborate statistics it takes time to embrace this almost inevitable (relatively new) concept in anaesthesia. Let us take a closer look at an example recently published in *Acta Anaesthesiologica Scandinavica* to explore fundamentals related to qualitative research. Drake‐Brockman et al. [1] used a qualitative descriptive phenomenological approach to further insights into our stagnant understanding of perioperative anxiety in children and their caregivers. Let us break this down.

Unlike *quantitative research*, whose holy grail is to understand the process and the HOW, *qualitative research* seeks to understand the WHY behind behaviours or beliefs and can better inform policy and lead to culturally sensitive solutions by its ability to provide a rich understanding of complex human issues. Perioperative anxiety is indeed a complex issue and what has eluded us in our understanding of this issue in the past may indeed be resolved more satisfactorily using a qualitative methodological approach.

Qualitative research allows us to study phenomena in natural contexts and to explore the deeper understanding of people's experiences and behaviours. Qualitative methods require researchers to embrace a paradigm, or lens, such as constructivism as used in this study. This constructivist lens is a way of seeing the world that differs from how, say, a scientist might approach the interpretation of statistics and numbers. The underlying premise for the constructivist lens is that reality is socially constructed and cannot be directly measured. Reality, in constructivism, is multiple and relative to, for example in this study, the patients and their families, healthcare professionals, and researchers and their context. This context is both shared (in the hospital environment) and unique, shaped by professional and personal perspectives and prior experience. Other common paradigms, or lenses, in qualitative research include interpretivism where researchers search within a cultural or historical interpretation lens of the social world to form an understanding of participants' experiences, based on Weber's *Verstehen*, or critical theory/inquiry, where reality is shaped by social, political, cultural, economic, ethnic and gender constructs over time [2].

Methodological approaches used by qualitative researchers could be narrative, exploring individual stories to describe people's lives, or could explore a shared culture of a group as in an ethnographic approach [2]. Drake‐Brockman et al.'s study used a phenomenological approach, which seeks to study the experience of the individual, truly emphasising their personal perspective and interpretation. Phenomenology studies the lived meaning of an experience, seeking to describe it, convey its meaning and to examine the contextual factors that influence it. In qualitative approaches, data is obtained through personal interactions and communication. *Words*, from interviews or group discussions, provide the data for qualitative studies. Data analysis therefore entails the sifting through those words and the concepts or ideas articulated to offer insights to issues and research questions not otherwise be obtainable by numerical data. Children's and their parents' voices and their lived experiences regarding perioperative anxiety have never previously been shared and highlighted in this way.

Qualitative methodology is slowly gaining some traction in medicine even though it has long been embraced by allied health specialties such as nursing and psychology. As early as 2001, the *Lancet* launched a short qualitative research series, recognising its potential to contribute to a broader understanding of medicine [3, 4]. Neurosurgeons have used qualitative methodologies to better understand equitable access to neurotechnology [5]. Phenomenology has been previously used to better understand patients' experiences living with chronic illness [6]. Respirologists have used this approach to better tailor a new healthcare programme to patients [7] and hospital organisations have used this methodology to help elucidate ways to mitigate staff burnout [8]. More recently, artificial intelligence (AI) may be contributing to this increased interest in qualitative research since it facilitates the transcriptions and analysis of interviews. Although it is not yet an acceptably widespread tool to use for coding and finding themes per se, AI‐facilitated transcripts may alleviate some of the workload in qualitative analysis [9].

Returning to our example published in this journal, the authors were able to successfully recruit an impressive number for qualitative research, of 24 pre‐teen children as well as their parents over a 2‐month period for participation in an average of 30‐min semi‐structured interviews. The research team also interviewed 20 staff, including volunteers, anesthesiologists, nurses, and non‐anesthesiologist members of the anesthesiology‐led care team (ACT). The rigour required in qualitative research is high and the work to transcribe all the interviews and then code the transcripts is extremely time‐consuming and exacting. Although some research teams elect to use a combination of inductive and deductive approaches for analysis, this research team chose to determine codes inductively, meaning that they combed through each sentence within the transcripts to identify patterns from their raw data set, instead of using predetermined patterns (a deductive approach). Coding inductively requires the research team to agree to the labelling of such patterns. Codes, and subcodes, are developed as interviews are completed and transcribed a labour‐intensive and iterative process.

Themes were identified from the codes upon which the research team agreed. These themes include the importance of uncertainty as a factor in the child's anxiety, expressed not only by the children, but also by the parents and the staff. Comfort and distraction were highlighted in these interviews to help mitigate some of this anxiety, whether provided by the parent, the staff or both. Staff expressed a discomfort with managing the perceived increased anxiety among their patients and their families, expressing their lack of preparedness, yet recognised the importance of their role to mitigate that anxiety for both the child and the parent. Trust in the healthcare team emerged as an important and overarching theme in alleviating that anxiety by all three groups interviewed. By understanding the WHY from this qualitative research clinicians are now in a better position to improve their clinical practice. For example, this study will hopefully lead to the institution investing and developing improved tools or workshops for staff to better prepare them to appropriately comfort and distract the anxious patient and parent. Time will tell if this qualitative approach to the understanding of the behaviours and beliefs will make a difference in outcome.

Qualitative research has the unique ability to contribute and further our understanding of other complex issues in our specialty. Taking a different angle to traditional science and potentially using AI to reduce the workload of analysis of this type of data and their analysis requires leaving our comfort zone of what we currently know and are very good at. The next step in our lives as clinicians may be to embrace qualitative research and its impact on our practice and our patients. Understanding what matters, not only how but why it matters contributes to a deeper understanding and perhaps acceptance of reality as a social construct in addition to ‘traditional’ science.

## Author Contributions

Natalie Buu contributed to this editorial by writing the orginal drafts which were revised by Thomas Engelhardt.

## Funding

This work was not funded.

## Conflicts of Interest

The authors declare no conflicts of interest.

## Data Availability

Data sharing not applicable to this article as no datasets were generated or analysed during the current study.
